# Mini-Trampoline Training Enhances Executive Functions and Motor Skills in Preschoolers

**DOI:** 10.3390/children12101405

**Published:** 2025-10-17

**Authors:** Mohamed Amine Ltifi, Yosser Cherni, Elena Adelina Panaet, Cristina Ioana Alexe, Helmi Ben Saad, Ana Maria Vulpe, Dan Iulian Alexe, Mohamed-Souhaiel Chelly

**Affiliations:** 1 University of Gafsa, Higher Institute of Sport and Physical Education of Gafsa, Gafsa 2112, Tunisia; mohamedltifi19@gmail.com; 2 Research Laboratory (LR23JS01) Sport Performance, Health & Society, Higher Institute of Sport and Physical Education of Ksar Said, University of Manouba, Tunis 2010, Tunisia; yossermrabet@hotmail.fr (Y.C.); mohamedsouhaiel.chelly@issep.uma.tn (M.-S.C.); 3 Department of Physical and Occupational Therapy, “Vasile Alecsandri” University of Bacau, 600115 Bacau, Romania; alexedaniulian@ub.ro; 4 Department of Physical Education and Sports Performance, “Vasile Alecsandri” University of Bacau, 600115 Bacau, Romania; zaharia.ana@ub.ro; 5 Heart Failure Research Laboratory (LR12SP09), Faculty of Medicine of Sousse, Farhat HACHED Hospital, University of Sousse, Sousse 4000, Tunisia; helmi.bensaad@rns.tn

**Keywords:** child development, executive functions, exercise therapy, physical fitness, preschool children, psychomotor performance

## Abstract

**Highlights:**

**What are the main findings?**

• Mini-trampoline training over 12 weeks improved motor abilities such as functional mobility, postural steadiness, and lower body strength in preschool children.
• The intervention also enhanced executive functions, particularly inhibitory control, supporting early cognitive development.

**What is the implication of the main finding?**

• This study provides new evidence from Tunisian preschoolers, highlighting the role of playful physical activity in fostering healthy growth.
• Findings suggest that mini-trampoline programs can be a safe, engaging, and effective approach for promoting both motor and cognitive skills in early childhood.

**Abstract:**

**Background**: Early childhood is crucial for motor and cognitive development, with physical activity playing a key role. Mini-trampoline exercises may offer an effective approach to enhance these domains. **Methods**: This study assessed the effects of a mini-trampoline program on executive functions and motor skills in Tunisian preschoolers. Fifty-four children (age 3.87 ± 0.47 years) participated in a 12-week intervention, divided into a control group (*n* = 27), following standard activities, and an experimental group (*n* = 27), engaging in mini-trampoline exercises. Pre- and post-tests measured motor skills like postural steadiness, balance, and coordination, as well as cognitive functions, including working memory (WM) and inhibition. **Results**: Significant improvements were observed in the experimental group for functional mobility, postural steadiness, lower body strength, and inhibition (*p* < 0.001), whereas the control group showed minimal changes. ANOVA revealed no significant group × time effects, except for a trend in postural steadiness (*p* = 0.062), suggesting a potential benefit of the intervention. **Conclusions**: These findings highlight the potential of mini-trampoline exercises to enhance motor skills and specific executive functions in preschoolers, supporting their overall development.

## 1. Introduction

Physical activity (PA) is recognized as a key factor in the growth and development of children [1]. In young children, PA provides multiple benefits, including weight control, motor development, and psychological well-being [2]. The prevalence of overweight and obesity among children is an increasing public health concern in Tunisia [3,4]. A study conducted in the metropolitan area of Tunis reported that 19.7% of elementary schoolchildren were overweight, and 5.7% were obese [5]. Moreover, excessive consumption of bread, snacks, and sugary drinks, as well as insufficient physical activity, were identified as contributing factors to overweight and obesity among school-aged children [5]. However, further studies are needed to confirm the relevance of these findings for preschool-aged children [4].

In 2019, WHO published PA guidelines for children under 5 [6]. The SUNRISE study in Tunisia showed low adherence, highlighting the urgent need to promote PA from preschool age [7]. The findings highlight the urgent need to promote healthy behaviors from preschool age, integrating PA and reducing screen time into public health initiatives [4]. Indeed, numerous studies, driven by advances in neuroscience and embodied cognitive science have examined the impact of PA programs (PAP) on children’s cognitive functions [8,9,10,11]. The majority of these investigations highlighted positive correlations between PA and improved cognitive abilities in children [8,12,13].

Executive function (EF) refers to the advanced cognitive ability to coordinate and regulate a set of cognitive processes in order to achieve a specific goal [14]. EF in preschool children is a key component of their individual development, as it can influence their future academic success [15] and social interaction skills [16]. Recent studies have shown that PA interventions can have beneficial effects on children’s EF [17,18,19]. Experimental research indicates that both acute and chronic aerobic exercises can effectively improve EF in children [20]. However, these findings have yet to be generalized to school or other natural environments, whether urban or rural [3], or across different gender (boys and girls), particularly for preschool-aged children. However, evidence suggests that not all PA modalities equally promote EF development [20].

Mavilidi et al. [21] found that integrating PA into a 4-week vocabulary program enhanced learning outcomes compared to traditional methods [21]. Further research is needed to strengthen the evidence on the effects of different PA practices on the development of EF, particularly during early childhood. Trampoline, often referred to as “air ballet,” is a form of gymnastics that is very popular among preschool children in China [22]. This activity involves acrobatic movements as children bounce on the trampoline, requiring constant adjustments in posture to adapt to changes in gravity with each jump [22]. It is a motivating practice for young children, combining enjoyment with motor skill development, creating an engaging and dynamic learning experience [22]. Trampoline activities have notable effects on children’s sensory organs and nervous system, enhancing aspects such as spatial awareness, vision, proprioception, and motor control as well as on their physical fitness, including strength, balance, and coordination [23,24]. A 12-week trampoline program (20 min/day) improved motor skills and balance in children [23,24].

In addition to these physical benefits, mini-trampoline exercises may also positively influence cognitive development, particularly executive functions. The continuous adjustments in posture, rhythm, and coordination during trampoline activities stimulate the prefrontal cortex [25] the brain region responsible for attention, inhibitory control, and working memory (WM). The dynamic nature of jumping tasks, which require children to anticipate, plan, and regulate their movements, creates opportunities for cognitive engagement and self-regulation. Moreover, the playful and emotionally stimulating context of mini-trampoline training can enhance motivation and arousal, both of which are known to support executive functioning in early childhood [13,20,26].

Moreover, mini-trampoline exercises offer a multi-faceted approach that positively impacts physical conditioning aspects such as balance, flexibility, and strength [23,24], as well as enhancing muscle coordination and spatial awareness [22]. Performing exercises on a flexible surface like a mini-trampoline may also reduce neuromuscular stress, potentially lowering the risk of injury [27], while simultaneously improving postural stability and jump performance [27,28]. These characteristics make mini-trampoline exercises beneficial and safe for younger children [29].

Trampoline exercises, popular among preschool children [24], are considered an ideal activity for enhancing both EFs and motor development. Given the lack of research on Tunisian preschoolers, this study aimed to assess the effects of mini-trampoline training on both EF and motor skills. Specifically, the objective was to assess the effects of a mini-trampoline PA program on EF (e.g., attention, inhibitory control) and motor skills (e.g., balance, coordination) in Tunisian preschoolers. We hypothesized that mini-trampoline training would significantly enhance EF and motor skills, offering an engaging way to support healthy growth.

## 2. Materials and Methods

### 2.1. Study Design

**Figure 1:**
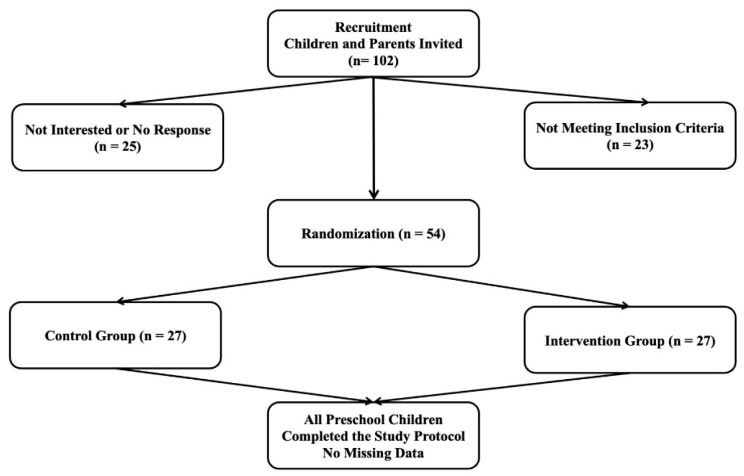
A schematic timeline of the study design following the initial recruitment.

As illustrated in Figure 1, the eligibility of preschool children was assessed, with a total of 102 children evaluated for participation in the study. Among them, 25 parents refused to allow their children to participate, and 23 children did not meet the inclusion criteria. In total, 54 children (26 boys and 28 girls) with a mean age of 3.87 ± 0.47 years were included in the study. The children were randomly assigned to either the experimental group (27 children; 14 boys) or the control group (27 children; 12 boys) using a computer-generated random number list.

### 2.2. Participants

Participants included fifty-four preschool children (age 3.87 ± 0.47 years; body mass 14.66 ± 2.58 kg; stature: 106 ± 6 cm). Participants were recruited from a nursery school École La Fontaine, located in the El Menzah 7 district of Tunis, Tunisia. The inclusion criteria for the children were strict, allowing only those without psychological or mental disorders or physical disabilities. This ensured that the study groups were comparable at the start of the trial. An introductory meeting was held with the school’s physical education teachers and the children’s parents to explain the research objectives and the procedures. Parents provided informed consent by signing a written form after being informed of the potential risks and benefits of the study. Parents also completed a detailed questionnaire regarding their children’s mental and physical health.

Participants were allocated into two groups (control group: age 3.99 ± 0.45 years; experimental group: age 3.76 ± 0.47 years) using the same computer-generated random number list. The assessors conducting the evaluations were blinded to group allocation. To maintain the confidentiality of the randomization, the process was carried out by an independent researcher not involved in the study. The control group followed standard preschool physical activities, including basic motor skills exercise (running, jumping, hopping, stretching routines, ball games, and free play typically) conducted during their regular physical education classes. However, the experimental group was engaged in rebound exercises on a mini-trampoline.

The 12-week mini-trampoline program was structured into three progressive phases to match the children’s gradual learning capacity. Each session lasted 20 min, conducted three times per week. During the first 4 weeks (phase 1), sessions focused on basic movements such as two-foot jumps, alternating foot jumps, and coordinated arm-leg movements (2 sets of 8–10 repetitions per exercise, with 30 s of rest). In weeks 5–8 (phase 2), the complexity increased with the introduction of lateral jumps, small rotations (90–180°), and balance-maintaining tasks on one foot (3 sets of 10–12 repetitions, with 20 s of rest). In the final 4 weeks (phase 3), more challenging exercises were included, such as multi-directional jumps, sequences with rhythm changes, and short combination tasks involving jumps and landing control (3 sets of 12–15 repetitions, with 15 s of rest). The progression was carefully monitored to ensure safety and adapt to each child’s abilities.

Descriptive data for the participants as age and anthropometric characteristics were presented in Table 1. All participants completed the study, and no dropouts were reported during the intervention period.

The participants contributed in a 12-week intervention, with assessments conducted before and after the program. They were tested twice in a pre-test followed by a post-test to measure motor skills as functional mobility (FM), postural steadiness (PS), upper and lower balance stability, work memory, Dexterity’s inhibition, work memory and inhibitory control.

The sample size for this study was calculated to ensure sufficient statistical power to detect within- and between-group differences in motor and EF measures. A two-way repeated measures ANOVA (Group × Time) was selected, with assumptions including a medium effect size (f = 0.25), an alpha level of 0.05, a power of 0.80, two groups (i.e., experimental and control), and two measurements (i.e., pre- and post-test). Using G*Power software (version 3.1.9.2, Heinrich Heine University, Düsseldorf, Germany), the minimum required sample size was 34 participants (17 per group). To account for potential dropout or non-compliance, the target was increased by 20%, resulting in a required sample size of 42 participants. This study included 54 children (27 per group), exceeding the minimum required sample size and ensuring robust representation for assessing the intervention’s effects on age, anthropometric characteristics, and gross and fine motor skills.

### 2.3. Ethical Approval

All procedures were approved by the Ethics Committee of the Faculty of Medicine of Sousse (CEFMS 121/2022). Written consent from parents and assent from participants were obtained before the study began. All participants, along with their parents or legal representatives, were fully informed about the study protocol and the potential risks and benefits involved.

### 2.4. Training Program: General Warm-Up and Mini-Trampoline Program

Each session consisted of a 10 min warm-up, followed by 10–15 min of mini-trampoline exercises, and a 5 min cool-down. Sessions were held three times per week for 12 weeks (Table 2).

The mini-trampoline training program, designed exclusively for the experimental group, features simple exercises tailored to the motor abilities of preschool children. Activities such as basic jumps with either feet, or coordinated arm movements are easy to perform and progressively evolve into more complex exercises, such as jumps with rotations, respecting the children’s capacity for gradual learning [22,23]. Short recovery periods (15 to 60 s) prevent excessive fatigue while maintaining participant engagement [27]. This program aligns with the physiological characteristics of children under five years old and adheres to fundamental training principles [30]. By promoting overall motor development, coordination, and balance, it ensures an age-appropriate, safe, and progressive approach that enhances skills without imposing undue stress [22,23].

### 2.5. Testing Procedures

Assessments were conducted over two days to minimize fatigue and ensure optimal performance. The order of completion between workshops is explained as follows:

• Day one
  ✓ Anthropometric measurements (height, body mass)
  ✓ Go/No-Go (Executive function test)
  ✓ One-leg balance
  ✓ Handgrip dynamometer (Upper body strength)
• Day two
  ✓ Mr Ant (Executive function test, working memory)
  ✓ Standing long jump (Lower body strength and mobility)
  ✓ Su-pine-Timed Up and Go (Mobility and posture)
  ✓ 9-Hole Pegboard Test (Fine motor skills)

#### 2.5.1. Anthropometry

Stature was measured to the nearest 0.1 cm using repositionable adhesive measuring tapes, with the child standing upright, barefoot, and with the head positioned according to the Frankfurt plane, following standardized procedures (Norgan 1988 [31]). Each measurement was performed twice, and if the difference exceeded 0.5 cm, a third measurement was taken. Body mass was assessed to the nearest 0.1 kg using a calibrated SECA 750 Viva scale (Hamburg, Germany), with the child in light clothing and without shoes. If the two readings differed by more than 0.25 kg, a third measurement was taken. Body mass index BMI (kg/m^2^) was calculated using WHO standards [32].

#### 2.5.2. Executive Function

Inhibition and WM, two key indicators of cognitive function, were assessed using the early years toolbox [33]. The French version of the games was translated verbatim into Arabic by a field worker, who was also a physical education teacher. A forward translation method was used to ensure accurate adaptation of the content. This Arabic version is currently undergoing validation among Tunisian preschool children. However, it has already been used in other studies conducted within the Tunisian context [7], confirming its acceptability and comprehensibility for this population [7]. The primary goal was for children to understand the task requirements during the practice phase. Each game took approximately 10 min to complete. Executive function tests assessed children’s behavioral impulse control and inhibition (Go/No-Go) and visuospatial WM [33]. The research assistant explained each game, which included a built-in practice period at the start. The tasks were performed in a quiet environment.

#### 2.5.3. Lower Body Strength (LBS) and Mobility: Standing Long Jump

In this test, a line is marked on the ground, and the child stands behind it. The child then jumps forward with both feet as far as possible. One practice jump is allowed, followed by two official attempts. The final score is calculated as the average of the recorded distances from the two attempts [34].

#### 2.5.4. Mobility and Posture: Supine-Timed up and Go

In this test, a line is drawn three meters from a wall, and a large target is marked on the wall at the child’s eye level. The child starts lying on their back with their feet on the line. At the signal, the child got up and ran to touch the target. They then returned to the starting line as fast as possible. One practice trial is allowed, followed by two official attempts [34].

#### 2.5.5. One-Leg Standing Balance Test

In this test, the child stands on one leg with their arms alongside their body. The free leg must not touch or wrap around the standing leg, though the arms can move for balance. Timing starts when the free leg lifts off the floor. The test is stopped if the child moves the standing leg or hooks the free leg around it. If the child maintains balance for 30 s, the test was repeated on the other leg. The time balanced on each leg was recorded, and the average time is used as the final score [34].

#### 2.5.6. Upper Body Strength (UBS): Hand Grip Dynamometer

This test assesses the ability of the hand and arm muscles to generate the tension and strength needed to maintain posture [34]. During the test, the child must squeeze the handgrip dynamometer (TKK 5825, Grip-A; Takei Scientific Instruments Co., Tokyo, Japan) with maximum force using their right hand for at least 3 s, ensuring that their arm does not touch their body. One practice trial and two test trials are performed with each hand (right and left), and the best value was used for the analysis [34].

#### 2.5.7. Manipulation: 9-Hole Peg-Board Test

This test evaluates the speed and accuracy of hand movements by having the child pick up nine pegs one by one, place them into a pegboard, and then remove them. Timing starts when the child begins the task and stops when the last peg is removed. The child should use the non-tested hand to stabilize the board, and the evaluator may also help by placing their hand on the child’s hand for added stability. The child completes two trials and two practice sessions, one with each hand. This test has proven to be reliable and valid for assessing fine motor skills in children [34].

### 2.6. Questionnaire

The questionnaire was given to the child’s parent or guardian (n = 54) and asked about the child’s usual sleep patterns, including sleep duration based on nighttime sleep. It also covered dietary diversity, eating habits, and household food insecurity. Socio-demographic information was collected using an adapted version of the WHO STEPS survey [35]. Questions about children’s movement behaviors were based on the 2017 Australian 24 h movement behavior guidelines for early years [34].

#### Center Information Questionnaire

The center information questionnaire was conducted through interviews with the kindergarten director. Like the parent and director questionnaires, the data collector read each question aloud, and participants gave verbal responses, which were recorded and entered directly into the questionnaire. The questionnaire collected information on the total number of eligible children at the center, those who consented to participate, and the timing of the daily nap.

### 2.7. Exercise Intervention

The study was conducted in three phases: (i) a pre-test evaluating EF and motor development, (ii) a 12-week intervention, and (iii) a post-test assessing EF and motor development. First, a pre-test was administered to evaluate the children’s baseline EF and motor development before the intervention. Over the following 12 weeks, both control and experimental groups attended the same classes and daycare services in kindergarten but engaged in different activities. The control group followed the usual PA program, while the experimental group participated in rebound exercises on a mini-trampoline. At the end of the 12-week intervention, a post-test assessing EFs and motor development was realized using the same tools as the pre-test. This post-test aimed to measure the impact of the intervention on EFs, motor development, and cognitive skills in preschool children.

For the experimental group, the sessions consisted of three weekly rebound exercise sessions, lasting between 10 and 30 min each, over the 12-week period. Each session began with a 10 min warm-up (Table 2). The rebound exercises were conducted under the supervision of two qualified physical education teachers, with each child using a personal mini-trampoline surrounded by safety nets. Additionally, two female supervisors, as required by the kindergarten’s internal regulations, were present to ensure the safety of the preschool-aged children. Safety recommendations from the American Academy of Pediatrics [36] were followed to prevent injuries.

### 2.8. Statistical Analysis

Data analysis was conducted using SPSS Statistics (Version 26) for Windows (SPSS, Inc., IBM Corp., Armonk, NY, USA) and expressed as means ± standard deviations. The comparison of the means of the two independent groups was performed using the non-parametric Mann–Whitney test. The analysis assessed within-group and between-group differences for the experimental and control groups across various motor and EF measures before and after the intervention. The normality of data distribution was verified using the Shapiro–Wilk test (*p* < 0.05), and sphericity was assessed using Mauchly’s Test. Homogeneity of variances was tested with Levene’s Test. A two-way repeated measures analysis of variance (2 × 2; group × time) was used to evaluate the effects of the intervention on each parameter. Post hoc comparisons were conducted where necessary to further explore significant interactions. Statistical significance was set at *p* < 0.05. The effect size was reported as Cohen’s *d* for within-group changes and partial eta squared (*ηp*^2^) for ANOVA results to determine the magnitude of effects. Cohen’s *d* was interpreted as small (0.2), medium (0.5), or large (0.8), while η*p*^2^ was classified as small (0.01 < η*p*^2^ < 0.06), medium (0.06 < η*p*^2^ < 0.14), or large (η*p*^2^ ≥ 0.14), based on Cohen’s guidelines [37].

## 3. Results

Table 1 presents the descriptive data for participants’ demographic characteristics and PA manipulation in both control and experimental groups. The average age in the control group is slightly higher compared to the experimental group. Specifically, the average age was 3.99 ± 0.45 years in the control group and 3.76 ± 0.47 years in the experimental group (*p* = 0.119). In terms of gender distribution, girls represent a comparable proportion in both groups, with 55.56% in the control group and 48.15% in the experimental group (Table 1). Significant differences were observed in body mass (*p* = 0.001) and body mass index (*p* = 0.008), whereas height did not differ significantly between groups (*p* = 0.446).

The statistical analysis results reveal notable changes between pre- and post-measurements in the experimental and control groups. In the experimental group, significant improvements were observed in FM (*p* < 0.001), postural steadiness (*p* < 0.001), and LBS (*p* < 0.001) (Table 3). In contrast, the control group demonstrated stability or marginal changes, particularly in LBS (*p* = 0.036). No significant improvements were observed in either group for UBS (experimental group: *p* = 0.787; control group: *p* = 0.425), dexterity (experimental group: *p* = 0.161; control group: *p* = 0.769), or WM (experimental group: *p* = 0.711; control group: *p* = 0.658). However, the experimental group exhibited a significant enhancement in inhibition (*p* < 0.001), while no change was observed in the control group (*p* = 0.230). These findings highlight the positive impact of the intervention on specific functional and motor abilities, particularly in the experimental group.

The results of the ANOVA group × time interaction reveal varying levels of significance and effect sizes across the measured variables. No significant interaction effects were observed for FM (*p* = 0.696), UBS (*p* = 0.986), dexterity (*p* = 0.988), or WM (*p* = 0.943), indicating minimal or no effect of the intervention in these areas. However, a marginal interaction was found for postural steadiness (*p* = 0.062, d = 0.369), suggesting a trend toward a significant effect. Similarly, LBS (*p* = 0.368) and inhibition (*p* = 0.366) showed moderate effect sizes but did not reach statistical significance. These findings reflect the nuanced impact of the intervention, with notable improvements in specific areas but no significant interaction effects when considering the group × time factor.

## 4. Discussion

The study revealed that the 12-week mini-trampoline exercise program significantly improved key motor skills such as FM, postural stability, and LBS, as well as inhibitory control of EFs in preschool children in Tunisia. However, ANOVA analyses did not reveal significant interactions for most variables. Given the lack of significant interactions, the results suggest trends rather than definitive effects. No significant interaction was observed for the majority of EFs and motor skills, but a marginal trend was noted for postural stability.

Numerous cross-sectional studies have investigated the relationship between PA and cognitive functions in both children and adults [38,39]. However, limited intervention studies have utilized PA, specifically rebound exercises on a mini-trampoline, to simultaneously enhance cognition and both gross and fine motor skills in preschool-aged children [10,40,41]. To the best of the authors’ knowledge, few intervention studies have examined the impact of mini-trampoline training on executive functions and motor skills in preschool children. This study provides an original contribution from Tunisia.

### 4.1. Effects of the 12-Week Mini-Trampoline Intervention on EFs

The observed improvement in inhibition within the experimental group aligns with findings suggesting that targeted PA programs can enhance inhibitory control, a core component of EFs [20]. The significant improvement might reflect the impact of the intervention on attentional focus and self-regulation, skills often stimulated through structured PAs. However, the absence of a significant group × time interaction indicates that these gains may not be exclusively attributable to the intervention, highlighting the need for further exploration of confounding factors.

For WM, the lack of significant improvement in both groups is consistent with research indicating that this EF develops gradually and may require more extended and diverse interventions to show measurable changes in preschool-aged children [26]. The delayed development of EFs in preschool-aged children is a well-documented phenomenon observed across various cultures and nations [10,11,30,42], and not unique to the Tunisian context. This developmental process requires repeated and adapted activities to achieve noticeable improvements. Moreover, the short duration of the intervention and the possible lack of cognitive challenge in the tasks might have limited its impact on WM.

The results revealed an improvement in inhibition among young children after 12 weeks of trampoline training. However, the study did not show a positive effect of this training on WM. This lack of effect may be attributed to the developmental trajectory of EFs, which requires repeated and tailored interventions to yield noticeable improvements. Additionally, socio-cultural and educational conditions may also play a role, affecting children’s ability to transfer acquired skills to more formal learning contexts [4,24]. The duration (12 weeks) and/or the intensity of the intervention may have limited the effects, although other studies have reported gains with shorter programs [24,39].

The relatively short duration of the intervention in the present study (12 weeks) may not have been the primary reason for the lack of significant improvement in certain EFs. While some authors [43], reported improvements in EF with an 8-week coordinative PA intervention (two 35 min sessions per week), others [39,44] observed benefits with just 4 weeks of task-integrated activities. These findings highlight that the type, intensity, and structure of the PA play a crucial role.

It has been reported that high-intensity PA may further promote cognitive development in preschool children [30]. A possible explanation for the lack of significant improvement in WM among Tunisian children in this study could be an insufficient intensity of PA to stimulate cognitive development [30]. However, a notable difference compared to some existing studies is that, while we did not observe significant improvements with moderate-intensity training, other research involving trampoline exercises has shown cognitive benefits even with similar or moderate intensity levels [21,29]. This suggests that, unlike our results, these studies observed improvements in executive functions despite similar intensity levels, which could indicate that other factors, such as the nature of the exercises, the duration of the intervention, or the specific characteristics of the samples, play a more significant role in the development of WM in preschool-aged children.

The type of PA is another key factor that can influence the effectiveness of an intervention. Over the past decade, the effects of various types of PA on EF have been explored [10]. Indeed, some studies have shown that aerobic exercises and coordinated PA are beneficial for EF development in preschool children [30,43]. In this context, our study also suggests that the type of physical activity plays a crucial role, as trampoline exercises showed benefits in certain areas but did not lead to significant improvements in others. This could be due to the specific nature of the task and the characteristics of the population studied, which may limit the generalizability of these results.

The positive effects of PA on EFs can be specific to the type of task, and the diversity in measurement methods presents challenges in consolidating the findings [34,39]. These tasks, specifically designed for children aged 3 to 5 years [11,45], are widely used to assess EF in young children in Western countries and China [46,47,48]. In line with these findings, our study also observed task-specific effects, as the Go/No-Go test showed positive results in the experimental group, suggesting that inhibitory control might be particularly responsive to trampoline-based PA. However, other tasks related to WM did not show similar improvements, which could be attributed to the nature of the exercises and the short duration of the intervention.

Children in the experimental group showed a greater increase in scores on the Go/No-Go test compared to those in the control group, suggesting that PA may be especially beneficial for enhancing inhibitory control in Tunisian preschoolers. Additionally, these results indicate that trampoline activity is highly engaging for children, which may contribute to observed improvements in their ability to control impulses and respond thoughtfully [24].

In this context, a growing body of evidence suggests that physical activity induces neuroplastic adaptations in the prefrontal brain regions, which play a central role in executive functions such as attentional control, inhibitory processes, and WM [26,49]. Regular engagement in moderate to vigorous physical activity promotes increased cerebral blood flow, positive regulation of neurotrophic factors such as BDNF (Brain-Derived Neurotrophic Factor), and synaptic plasticity, particularly in the dorsolateral and ventromedial prefrontal cortex [50]. These adaptations are especially important during early childhood, a period marked by rapid brain development and high neural plasticity. Therefore, the improvements observed in inhibitory control in our study may reflect not only behavioral training but also underlying neural changes induced by trampoline-based activity, which engages both motor coordination, arousal, and sustained attention.

This study is the first in Tunisia to examine the effects of mini-trampoline training on EF in preschool children, highlighting its originality. Further studies with larger samples and longitudinal, multi-center designs are needed to confirm these findings and explore the influence of factors such as socio-economic status, sex, and baseline motor skills.

### 4.2. Effects of the 12-Week Mini-Trampoline Intervention on Gross and Fine Motor Skills

The 12-week mini-trampoline intervention showed significant improvements in several aspects of gross motor skills in the experimental group compared to the control group. The most notable gains were observed in FM, PS, and LBS, highlighting the effectiveness of this specific PA. In contrast, no significant changes were observed in fine motor skills or UBS, with both groups remaining stable on these parameters. The results of the ANOVA group × time interaction revealed varying levels of significance across the measured variables. No significant interaction effects were observed for FM, UBS, dexterity, or WM, indicating that the intervention had minimal or no impact when considering the group × time interaction. However, a marginal interaction was noted for PS, showing a trend toward a significant effect. Similarly, LBS and inhibition showed moderate effect sizes but did not reach statistical significance.

Nevertheless, within-group analyses indicated notable improvements in certain gross motor and executive function components (particularly FM, PS, LBS, and inhibition) among children in the experimental group.

The results of this study demonstrate the effectiveness of mini-trampoline exercises in improving certain gross motor skills, notably balance, mobility, and LBS. These within-group improvements align with the findings of Giagazoglou et al. [23] who reported significant gains in motor performance following a similar training protocol. Similarly, Arabatzi [40] highlighted the crucial role of trampoline activities in physical development across various contexts, even when group × time effects are not statistically significant.

The improvements observed in the experimental group can be attributed to the continuous activation of the proprioceptive and sensory systems (vestibular, visual, and somatosensory) during the exercises. These stimulations promote real-time postural adjustments, thereby enhancing neuromuscular control and balance stability [27,28]. The engagement of trunk and lower body muscles also contributes to muscular coordination and functional strength, while the repetitive nature of the exercises supports motor learning and optimizes movement patterns [15,23]. However, the lack of significant changes in UBS and fine motor skills can be explained by the targeted nature of the exercises. Indeed, mini-trampoline activities primarily focus on lower body stability and coordination. As suggested by Di Stefano et al. [51] and Mandelbaum et al. [52], although training on unstable surfaces strengthens the trunk and lower limb muscles, it may not provide sufficient stimulus for neuromuscular adaptations in upper body muscles. Moreover, fine motor skills, such as precision and hand movement speed, require specific stimulation, which is not addressed in trampoline rebound exercises [24].

Our results showed no significant effect of the intervention on fine motor skills, likely due to the mini-trampoline PAP primarily targeting gross motor skills development [24]. The lack of specific stimulation for precise hand and finger movements may account for the absence of observed improvements. Further research is needed to explore the potential impact of PA, particularly mini-trampoline exercises, on fine motor skills.

The lack of significant group × time interactions in this study may be attributed to the biological and neuromuscular development stage of preschool-aged children, whose sensory and proprioceptive systems are still maturing [20,24,26]. This developmental stage is characterized by rapid growth but also high variability, making adaptations to physical interventions less pronounced compared to older children or adults [42]. Gross motor skills, such as balance and coordination, are more responsive to physical interventions as they involve large muscle groups, while fine motor skills like dexterity require more targeted and prolonged stimulation [27,28]. Additionally, the physiological responses to PA in preschool children differ significantly from those of prepubescent or adult populations due to differences in muscular, hormonal, and cardiovascular development [53]. These factors likely influence the magnitude of adaptations observed in this age group [53].

Additionally, the high variability in motor and cognitive development among children in this age group may have affected the consistency of responses, reducing the likelihood of detecting significant effects in group analyses [54]. Lastly, the intervention’s duration may have been insufficient to produce lasting changes in areas such as fine motor skills and WM, which demand extended cognitive and sensory integration [55].

From the perspective of Dynamic Systems Theory, motor development emerges from the continuous and nonlinear interaction between the child, the task, and the environment [56,57]. Thus, improvements in motor performance depend not only on the training stimulus but also on each child’s intrinsic characteristics (e.g., maturation, motivation, coordination) and the context in which activities are performed. This theoretical framework helps explain the variability of responses observed in our study, suggesting that, even in the absence of significant group-level effects, meaningful individual adaptations may occur through the self-organization of the motor system during practice and interaction with the environment.

Finally, the duration of the intervention may have been insufficient to produce significant changes in areas such as WM or dexterity, which require not only physical stimulation but also prolonged cognitive and sensory integration [20]. These areas are known to be influenced by repeated, tailored, and longer-term interventions [24].

### 4.3. Study Limitations

The absence of objective PA measurements (e.g., accelerometers) and a possibly non-optimized program may have limited the results. Participants were recruited from a single private school in Tunis, which may limit the generalizability of the findings. Future studies should incorporate objective intensity measurements, refine program designs, recruit larger and more diverse samples, and include multiple schools to maximize the impact and external validity.

### 4.4. Practical Applications

Given the accessibility and engaging nature of mini-trampoline activities, they can be integrated into preschool physical education programs to support fundamental movement skills in a playful and motivating environment. Despite the absence of significant effects on WM and fine motor skills, the results indicate that trampoline-based exercises primarily benefit balance, coordination, and inhibitory control. Educators and coaches can implement structured trampoline sessions to foster physical and cognitive development while ensuring safety and age-appropriate progressions. Future research should explore long-term adaptations and consider combining mini-trampoline training with other motor-cognitive activities to maximize developmental benefits in young children.

## 5. Conclusions

This study provides valuable insights into the benefits of a mini-trampoline physical activity program for Tunisian preschool children, highlighting improvements in motor skills and certain executive functions, particularly inhibitory control. Significant gains in functional mobility, postural steadiness, and lower balance stability underscore the program’s effectiveness in promoting physical development in this age group. However, no notable improvement was observed in WM, upper balance stability, or fine motor skills, suggesting that the intervention primarily supports gross motor development rather than fine motor skills or complex cognitive functions. The lack of significance in the group × time interactions can be explained by several factors related to the biological and neuromuscular development of preschool-aged children, as their sensory and motor systems are still maturing. These findings help bridge the research gap regarding the impact of physical activity on motor and cognitive development in young Tunisian children, enriching our understanding of trampoline-based programs. Longer and more diverse studies are needed to further explore these effects.

## Figures and Tables

**Table 1:** Descriptive data for participants as age, anthropometric characteristics and gender.

Variables	Experimental Group (*n* = 27)	Control Group (*n* = 27)	*p* Value	Overall (*n* = 54)
Age (years)	3.76 ± 0.47	3.99 ± 0.45	0.119	3.87 ± 0.47
Body mass (kg)	17.13 ± 2.21	15.44 ± 1.61	0.001	16.28 ± 2.10
Height (cm)	105.23 ± 6.13	106.72 ± 6.03	0.446	105.98 ± 6.07
Number of girls (n, %)	13 (48.15)	15 (55.56)		28 (51.86)
Body mass index (kg/m^2^)	15.59 ± 2.47	13.72 ± 2.38	0.008	14.66 ± 2.58

**Table 2:** Details of general warm-up and mini-trampoline session protocol performed by participants.

Session Component	Objective	Mini-Trampoline Exercises	Details
Total session	Prepare and optimize overall physical readiness		Duration: 20–30 min Frequency: 3 sessions per week for 12 weeks
Warm-up: 10 min	Prepare the body for exercise, activate muscles, and reduce injury risks	**1.** Basic jumps on the mini-trampoline: • Position: Feet parallel, shoulder-width apart (30 s × 5 repetitions, 15 s rest) **2.** Dynamic stretches on the mini-trampoline: • Arms: Circular movements • Legs: Knee raises and heel kicks (10 repetitions each)	Activates major muscle groups and improves joint mobility, ensuring safe participation in the main session
Main session: 10–15 min	Develop motor coordination and neuromuscular efficiency using the mini-trampoline	***Exercise 1:*** Basic jumps • 2 min × 2 sets on the mini-trampoline • Rest: 1 min ***Exercise 2:*** Knee raises with jumping • 1 min × 3 sets on the mini-trampoline •Rest: 30 s ***Exercise 3:*** Lateral jumps • 1 min × 2 sets on the mini-trampoline • Rest: 1 min ***Exercise 4***: Arm-leg coordination jumps • 1–2 min on the mini-trampoline • Rest: 30 s	All activities focus on the mini-trampoline to enhance coordination, stability, endurance, and proprioception
Cool-down: 5 min	Gradually lower heart rate and relax muscles after mini-trampoline activities	**1.** Light jumps on the mini-trampoline: 2 min **2.** Static stretches off the mini-trampoline: • Quadriceps: Hold 10–15 s per side • Hamstrings, back, and arms	Facilitates muscle recovery, reduces post-exercise soreness, and improves long-term flexibility

**Table 3:** Overall scores for the gross and fine motor skill.

Variables	Experimental Group (*n* = 27)					Control Group (*n* = 27)					ANOVA	
	Pre	Post	Δ (%)	*p*	*d* (Cohen)	Pre	Post	Δ (%)	*p*	*d* (Cohen)	*p*	*d* (Cohen)
FM (s)	5.35 ± 1.07	5.19 ± 1.07	−3.07 ± 0.96	<0.001	0.10	5.26 ± 1.26	5.26 ± 1.01	0.18 ± 1.12	0.799		0.696	0
PS (s)	12.93 ± 8.72	19.69 ± 13.32	53.22 ± 9.39	<0.001	−0.61	12.63 ± 6.72	12.63 ± 6.85	−0.01 ± 4.69	1.000		0.062	0.369
LBS (cm)	50.89 ± 21.63	58.41 ± 24.09	15.31 ± 2.86	<0.001	−0.48	48.70 ± 18.41	49.00 ± 18.38	0.40 ± 3.96	0.036	−0.01	0.368	0
UBS (kg)	7.13 ± 2.75	7.15 ± 2.69	0.70 ± 5.05	0.787		7.43 ± 2.70	7.46 ± 2.73	0.40 ± 3.69	0.425		0.986	0
Dexterity (s)	38.93 ± 8.05	39.00 ± 8.09	0.17 ± 0.80	0.161		41.22 ± 10.53	41.24 ± 10.51	0.06 ± 0.87	0.769		0.988	0
Inhibition (a.u.)	0.68 ± 0.21	0.75 ± 0.22	11.73 ± 3.00	<0.001	−0.01	0.68 ± 0.22	0.68 ± 0.22	0.57 ± 2.06	0.230		0.366	0
WM (a.u.)	1.86 ± 0.62	1.87 ± 0.61	0.74 ± 6.66	0.711		1.80 ± 0.82	1.79 ± 0.78	0.40 ± 7.80	0.658		0.943	0

Note: all data are showed as mean ± standard deviation, FM: functional mobility “Mobility and posture: Supine-timed up and go”. LBS: lower body strength and mobility “standing long jump”. PS: postural steadiness “one-leg standing balance test”. UBS: upper body strength “strength hand grip dynamometer”. WM: Working Memory. “Mr Ant”. ANOVA (Group × Time interaction).

## Data Availability

The original contributions presented in this study are included in the article. Further inquiries can be directed to the corresponding author.

## Abbreviations

The following abbreviations are used in this manuscript:

EF	Executive Function
FM	Functional Mobility
LBS	Lower Body Strength
PA	Physical Activity
PAP	Physical Activity Program
PS	Postural Steadiness
UBS	Upper Body Strength
WM	Working Memory
WHO	World Health Organization
